# Supplementary material for: Cultures of Human Skin Mast Cells, an Attractive In Vitro Model for Studies of Human Mast Cell Biology
Source: Cells. 2024 Jan 2;13(1):98. doi: 10.3390/cells13010098 (PMC10778182; doi:10.3390/cells13010098)
Supplement: Supplementary file 1 [file cells-13-00098-s001.zip › cells-2764371-supplementary.pdf]

Supplementary Tables

Table S1. Transcript levels in reads from an Ampliseq analysis of freshly isolated MCs from foreskin and from purified MCs from breast skin and foreskin MCs cultured for 2-3 weeks for proteoglycans and other carbohydrate related functions.

	Freshly isolated cells			Cells cultured for 2-3 weeks			
	Foreskin (Male)			Foreskin (Male)		Breast skin (Female)	
**Proteoglycans and other carbohydrate related functions**							
SRGN	5850	7939	8529	4557	3642	3982	3936
HS3ST1	46	19	54	253	292	91	367
HS6ST1	293	108	202	346	419	292	478
HSPG2	198	127	55	88	84	70	82
GALNT6	59	68	50	57	76	63	60
CHST15	10	11	1	29	61	30	16
RENBP	99	63	82	127	129	138	176
GBE1	139	151	199	208	191	161	156
GNS	135	270	217	394	391	296	332
SDC3	87	74	44	103	94	157	97
B3GNT5	79	100	152	40	44	18	29
B4GALT5	129	255	321	48	47	43	45
PRG2	13	29	15	165	204	126	77
NDST1	65	54	33	85	113	112	108
EXT1	119	91	87	147	165	168	177
EXTL3	65	25	37	58	76	65	83
ST8SIA1	33	14	31	78	61	49	67
GNPTAB	168	83	68	358	334	297	383
LGALS3	679	885	830	396	410	484	487
GM2A	616	367	631	898	1162	924	1297

Table S2. Transcript levels in reads from an Ampliseq analysis of freshly isolated MCs from foreskin and from purified MCs from breast skin and foreskin MCs cultured for 2-3 weeks for solute carriers.

	Freshly isolated cells			Cells cultured for 2-3 weeks			
	Foreskin (Male)			Foreskin (Male)		Breast skin (Female)	
**Solute Carriers**							
SLC1A5	344	376	409	142	149	162	118
SLC6A8	30	72	77	1	0.3	0.5	2
SLC7A8	2	2	2	118	144	190	151
SLC8A3	47	10	29	89	85	98	102
SLC9A1	210	150	208	120	113	118	128
SLC16A3	52	181	131	173	165	210	162
SLC18A2	698	702	1733	1818	1662	1044	1819
SLC22A17	8	8	2	83	126	249	120
SLC24A3	71	35	59	100	105	123	104
SLC25A44	467	189	415	33	47	40	38
SLC29A1	102	70	73	97	91	95	66
SLC30A1	57	50	42	146	212	310	298
SLC40A1	68	58	129	17	15	41	78
SLC43A3	352	387	355	359	368	336	328

SLC44A1	118	203	137	235	228	248	213
SLC45A3	200	70	213	267	304	240	250
SLCO2B1	154	164	205	203	250	159	191
SLCO3A1	37	32	30	36	41	48	44
SLCO4A1	52	85	50	0.3	0.2	0.2	0.1
**Lipid transporters and their receptors and lipid metabolic enzymes**							
APOE	58	53	50	0.9	3	1	0.6
FADS2	105	119	132	1276	1735	1227	1169
LRP4	148	131	165	56	54	46	93
**S100 proteins**							
S100A11	322	749	356	647	682	644	692

Table S3. Transcript levels in reads from an Ampliseq analysis of freshly isolated MCs from foreskin and from purified MCs from breast skin and foreskin MCs cultured for 2-3 weeks for calcium, sodium and potassium channels-transporters and other cell surface proteins.

	Freshly isolated cells			Cells cultured for 2-3 weeks			
	Foreskin (Male)			Foreskin (Male)		Breast skin (Female)	
**Calcium, Sodium and Potassium channels-transporters**							
CACNA2D2	84	59	97	29	49	121	99
ATP1B1	204	187	257	81	64	30	76
**Other Cell Surface proteins**							
GPNMB	543	467	715	478	532	112	338
TMEM246	70	102	99	135	174	110	102
EMP1	333	517	137	3	0.5	1	2
DCBLD2	78	36	83	371	335	452	541
TJP2	60	110	50	40	42	30	4
MPP1	211	203	126	316	308	303	242
NEO1	49	48	43	13	13	8	11
ITM2A	93	135	64	40	45	79	89
KCTD20	82	166	150	196	151	128	154
EPB41L1	176	98	93	390	339	293	323
SEMA7A	164	100	203	335	460	699	460

Table S4. Transcript levels in reads from an Ampliseq analysis of freshly isolated MCs from foreskin and from purified MCs from breast skin and foreskin MCs cultured for 2-3 weeks for Siglecs.

	Freshly isolated cells			Cells cultured for 2-3 weeks			
	Foreskin (Male)			Foreskin (Male)		Breast skin (Female)	
**Siglecs**							
SIGLEC6	484	289	515	1281	1436	1361	1490
SIGLEC8	129	33	108	130	198	181	243
SIGLEC5	16	10	21	19	26	11	46
SIGLEC9	19	25	37	41	50	91	44

Table S5. Transcript levels in reads from an Ampliseq analysis of freshly isolated MCs from foreskin and from purified MCs from breast skin and foreskin MCs cultured for 2-3 weeks for olfactory and other receptors.

	Freshly isolated cells			Cells cultured for 2-3 weeks			
	Foreskin (Male)			Foreskin (Male)		Breast skin (Female)	
**Olfactory receptors**							
OR2A4	18	17	19	94	67	93	47

OR2A7	7	5	12	28	25	33	23
OR2G3	3	7	4	11	20	6	10
OR2G2	3	7	5	6	8	3	2
**Other Receptors**							
LRP4	148	131	165	56	54	46	93
STAB1	22	14	17	24	28	41	46
GPR141	80	79	127	53	69	91	67
FZD1	28	24	25	59	49	45	83
FZD5	46	30	51	3	4	1	3
SCARF1	22	21	27	20	22	32	20
GPR183	250	216	167	2	0	3	3
LDLR	263	535	152	226	285	179	216
VDR	10	22	19	77	109	115	105
KLRG1	202	96	241	422	368	368	366
TSPAN4	86	69	71	90	93	106	156
DRD2	35	19	56	71	57	94	47
CNRIP1	113	74	81	241	224	382	124
AMHR2	59	256	57	86	104	85	101
ADORA3	16	3	5	14	20	10	12

Table S6. Transcript levels in reads from an Ampliseq analysis of freshly isolated MCs from foreskin and from purified MCs from breast skin and foreskin MCs cultured for 2-3 weeks for cell signaling components.

	Freshly isolated cells			Cells cultured for 2-3 weeks			
	Foreskin (Male)			Foreskin (Male)		Breast skin (Female)	
**Cell Signaling components**							
ARHGAP18	299	345	402	621	532	521	617
RGS13	281	181	339	354	252	352	435
AGAP1	226	200	244	256	288	334	280
BTK	155	66	125	469	352	436	426
LAT	290	352	371	413	490	380	513
GCSAML	468	663	589	1268	1019	926	1231
DUSP1	11964	2666	4799	15	14	26	18
DUSP3	132	143	189	32	35	27	43
DUSP4	146	488	96	318	327	460	449
DUSP6	728	951	624	346	362	451	390
DUSP7	254	137	247	75	104	87	92
DUSP14	130	433	229	99	86	123	143
DUSP23	52	36	44	111	132	112	121
LCP2	427	252	303	730	775	972	919
PPM1H	118	37	53	131	156	132	135
RASGRP4	118	48	126	238	281	297	283
ARHGEF40	104	91	100	72	93	61	79
GNAI1	98	28	22	136	147	153	147
PIK3R3	43	40	31	35	86	48	72
MAPK12	42	22	36	30	45	47	64
RAB32	40	38	46	93	82	72	81
RAB34	60	54	45	55	57	64	51
RAB27B	277	87	143	587	654	679	712
RIN2	54	45	46	4	2	3	3
TIAM1	123	98	135	268	344	480	510

ARHGAP6	28	27	27	149	148	201	148
ARHGAP18	299	345	402	61	532	521	617
PLK1	2	1	1	117	95	60	52
PDE3A	44	17	34	29	46	61	58
ITK	78	395	128	48	46	31	29
TIE1	27	28	13	50	42	35	33
RUSC2	40	48	32	43	48	48	49
PPM1H	118	37	53	131	156	132	135
GMPR	88	52	73	503	381	516	410
LPCAT2	202	95	103	146	185	166	156
CDK15	192	53	106	122	127	112	118
EVPL	327	168	472	55	55	54	75
SOX13	105	144	135	77	86	112	97
GNAQ	127	108	108	134	141	163	164
DAPK1	75	121	111	137	140	148	116
FHL3	42	41	53	24	32	15	14
NDRG2	191	158	230	98	116	97	103
FSCN1	211	230	268	72	123	30	52
TYROBP	735	543	949	1360	1464	1229	1936
TNIK	261	278	384	258	278	220	275
NREP	60	45	64	63	67	89	53
SDCBP	278	240	91	270	267	262	235
ALS2	145	80	164	195	197	192	153
TESPA1	528	272	558	1428	1632	1600	1717
DLC1	690	419	998	597	546	560	542
LAX1	207	172	206	292	296	323	287
CALB2	98	295	125	320	450	301	351
NCS1	31	185	35	61	51	48	63
MLPH	200	152	307	161	177	153	205

Table S7. Transcript levels in reads from an Ampliseq analysis of freshly isolated MCs from foreskin and from purified MCs from breast skin and foreskin MCs cultured for 2-3 weeks for other enzymes.

	Freshly isolated cells			Cells cultured for 2-3 weeks			
	Foreskin (Male)			Foreskin (Male)		Breast skin (Female)	
**Other enzymes**							
MAOA	9	10	0.7	765	721	924	848
MAOB	200	105	124	557	523	531	462
ZNRF1	102	55	79	165	153	179	157
NDFIP2	118	108	103	438	393	487	391
AGPAT9	213	335	338	142	112	152	99
BLVRA	77	31	15	236	280	226	262
PADI2	59	47	66	60	103	68	119
PADI4	0	0	0	0.5	0.2	0.4	0.6
INPP1	35	39	20	33	24	34	26
CHN2	64	86	40	51	45	56	48
FADS1	25	14	6	254	360	230	207
DCAF12	55	83	79	92	107	104	103
ANKRD9	96	109	185	44	20	37	20
ENPP2	107	86	57	25	22	24	53
MAT2A	254	142	217	101	164	173	133
ALAS1	155	90	202	561	785	813	864

MBOAT7	291	324	541	376	438	337	482
GLUL	2816	5447	2432	3182	3873	3049	3208
TRIM63	146	162	60	143	120	57	87
ALDH1A1	100	50	33	448	359	316	584
TYMS	4	7	10	713	477	553	321

Table S8. Transcript levels in reads from an Ampliseq analysis of freshly isolated MCs from foreskin and from purified MCs from breast skin and foreskin MCs cultured for 2-3 weeks for components of vesicle transport.

	Freshly isolated cells			Cells cultured for 2-3 weeks			
	Foreskin (Male)			Foreskin (Male)		Breast skin (Female)	
**Vesicle transport**							
STX3	149	47	100	259	298	267	324
STX11	196	327	268	69	68	60	68
DYNLL1	457	756	669	600	764	666	765

Table S9. Transcript levels in reads from an Ampliseq analysis of freshly isolated MCs from foreskin and from purified MCs from breast skin and foreskin MCs cultured for 2-3 weeks for cytoskeletal, nuclear, enamel and angiogenesis related proteins.

	Freshly isolated cells			Cells cultured for 2-3 weeks			
	Foreskin (Male)			Foreskin (Male)		Breast skin (Female)	
**Cytoskeleton**							
KRT19	162	160	174	167	215	246	190
SHROOM1	40	21	28	20	35	47	50
MYO10	85	110	136	98	110	123	146
TUBB3	18	49	38	216	313	253	217
TPPP3	95	179	120	0	0	0.1	0.1
DBN1	66	65	31	4	2	6	13
PDLIM7	124	179	61	47	64	50	42
GSN	205	198	39	104	149	198	124
TNS1	848	683	567	361	444	468	364
CAPG	507	567	727	1880	2312	1969	2168
CTTNBP2	138	116	106	367	467	467	494
SPTB	82	45	89	33	42	48	26
**Nuclear located proteins**							
SLFN5	208	65	129	236	241	195	325
LMNA	4302	6983	5755	462	496	503	467
**Enamel formation**							
TUFT1	47	67	71	2	4	2	4
**Angiogenesis promoting and eNOS activity**							
ENG	113	178	83	5	4	6	7
SDPR	290	49	48	510	466	525	540

Table S10. Transcript levels in reads from an Ampliseq analysis of freshly isolated MCs from foreskin and from purified MCs from breast skin and foreskin MCs cultured for 2-3 weeks for nuclear proteins.

	Freshly isolated cells			Cells cultured for 2-3 weeks			
	Foreskin (Male)			Foreskin (Male)		Breast skin (Female)	
**Nuclear proteins**							
PHLDA1	154	220	28	82	89	143	62
AHNAK2	139	75	120	119	164	91	104

Table S11. Transcript levels in reads from an Ampliseq analysis of freshly isolated MCs from foreskin and from purified MCs from breast skin and foreskin MCs cultured for 2-3 weeks for extracellular matrix, oncogenes, endogenous retroviral and proteins of unknown function.

	Freshly isolated cells			Cells cultured for 2-3 weeks			
	Foreskin (Male)			Foreskin (Male)		Breast skin (Female)	
**Extracellular matrix**							
COL13A1	72	34	63	104	101	91	165
COL18A1	114	198	145	95	86	78	74
FREM1	34	14	36	36	54	43	34
FERMT2	92	103	31	72	66	66	53
EMILIN2	700	750	1159	147	161	161	269
**Oncogenes**							
DLC1	690	419	998	597	546	560	542
FES	60	56	48	90	115	118	148
ETS2	395	965	394	32	42	31	30
**Endogenous Retrovirus**							
ERVFRD-1	174	85	156	118	189	229	296
**Proteins of unknown function**							
FAM46A	311	168	668	137	81	131	181
FAM129B	520	403	739	544	598	501	634
OAF	209	176	310	110	122	64	138
VWA5A	1440	941	2052	1954	1981	2054	2531
TMEM246	70	102	99	135	174	110	102
NAV1	128	44	64	96	134	167	106
